# Immune Checkpoint Inhibitor-Induced Central and Peripheral Neurotoxicity: Case Series and Literature Review

**DOI:** 10.7759/cureus.95344

**Published:** 2025-10-24

**Authors:** Shuvangee Dhar, Naresh Mullaguri, Sanjeev Sivakumar, Eduardo Cortez-Garcia

**Affiliations:** 1 Neurology, Prisma Health-Upstate/University of South Carolina School of Medicine Greenville, Greenville, USA; 2 Medicine/Neurology/Neurocritical Care, Clemson University School of Health Research, Clemson, USA; 3 Medicine/Neurology/Neurocritical Care, University of South Carolina School of Medicine Greenville, Greenville, USA; 4 Neurocritical Care, Prisma Health Greenville Memorial Hospital, Greenville, USA; 5 Neurology/Neurocritical Care, Prisma Health-Upstate/University of South Carolina School of Medicine Greenville, Greenville, USA; 6 Neurology/Neuromuscular Medicine, Prisma Health-Upstate/University of South Carolina School of Medicine Greenville, Greenville, USA

**Keywords:** central neurotoxicity, encephalitis, immune-checkpoint inhibitor, myasthenia gravis, peripheral neurotoxicity

## Abstract

Immune checkpoint inhibitors (ICIs) have been instrumental in the management of primary thoracic and non-thoracic metastatic malignancies, making them a mainstay of cancer therapy due to their long-term clinical benefits. However, they are also associated with several immune-related adverse events (irAEs), including neurological irAEs (nirAEs). Central neurotoxicities induced by ICI use include meningoencephalitis, focal encephalitis, myelitis, and aseptic meningitis, whereas peripheral neurotoxicities include ICI-associated myopathy, myasthenia gravis, and inflammatory neuropathy. The occurrence of irAEs and nirAEs can have long-term effects on survival due to the development of cognitive impairment, aphasia, physical debility, and potential dependence on tracheostomy and gastrostomy in the long term, and should be anticipated at any time following ICI therapy. Discontinuation of ICIs and administration of high-dose corticosteroids are first-line treatment options; however, cessation of ICIs increases the risk of malignancy progression. Rechallenging with ICIs therefore warrants a careful risk-benefit analysis. In this case series, we present four cases of central and peripheral neurotoxicity induced by ICI therapy to illustrate the diverse clinical presentations, prognoses, and outcomes, and to advance the discussion on discontinuation and rechallenging of ICIs in cancer patients.

## Introduction

Immune checkpoint inhibitors (ICIs) have been instrumental in the management of metastatic cancer, especially in malignancies such as melanoma, breast cancer, ovarian cancer, and other primary thoracic malignancies, including small-cell and non-small cell lung carcinoma [1,2]. ICIs primarily work by inhibiting cytotoxic inhibitory signaling pathways that tumor cells exploit to evade immune detection, such as cytotoxic T-lymphocyte-associated protein 4 (CTLA-4), programmed cell death protein 1 (PD-1) on T cells, and programmed cell death ligand 1 (PD-L1) on tumor cells [3,4]. CTLA-4 inhibitors include ipilimumab and tremelimumab; PD-1 inhibitors include pembrolizumab, nivolumab, toripalimab, and cemiplimab; and PD-L1 inhibitors include atezolizumab, avelumab, cosibelimab, and durvalumab [3,4]. The immunological checkpoints represent a group of inhibitory and stimulatory pathways that regulate immune cell activity and maintain a balance between pro-inflammatory and anti-inflammatory signals under homeostatic conditions [5]. ICI activity, specifically targeting CTLA-4, PD-1, and PD-L1, has been approved by the FDA and has become a widely used immunotherapeutic approach in the last decade [5]. The long-term clinical benefits of ICIs and their improvement in overall survival in patients with multiple tumor types have made them a mainstay of cancer therapy alongside traditional treatment modalities such as surgery, chemotherapy, and radiation [6]. The estimated percentage of cancer patients in the United States eligible for ICIs increased from 1.54% in 2011 to 43.63% in 2018, while the proportion of responders rose from 0.14% in 2011 to 12.46% in 2018 [7], indicating the growing prevalence of ICI use.

Although ICI therapy provides significant clinical benefits, it is also associated with several adverse effects. PD-1/PD-L1 and CTLA-4 are widely expressed across various tissue types, and their downregulation can trigger a broad array of autoimmune toxicities [1]. Common immune-related adverse events (irAEs) include gastrointestinal, pulmonary, endocrine, and dermatologic toxicities [8], with variable frequencies ranging from 15% to 90% [2]. Neurologic irAEs (nirAEs) are relatively rare (approximately <5%); however, their potential severity and the resulting interruptions to cancer treatment warrant particular attention [9]. The onset of nirAEs following the first administration of immunotherapy can range from 2 to 4 months, although delayed presentations of up to 68 weeks have also been reported [10]. The peripheral nervous system is more commonly affected, with pathologies such as ICI-associated myopathy, myasthenia gravis (MG), and inflammatory neuropathies (polyradiculoneuritis and sensory ganglionopathy), compared with central nervous system pathologies such as meningitis, myelitis, and encephalitis [11]. Neuromuscular disorders such as MG are often more frequent, accounting for nearly 13.5% of all nirAEs, and typically present earlier than central neurotoxicities [4]. Although MG is a less common sequela compared with ICI myopathy and neuritis, it is associated with the highest morbidity and mortality rates, occurring more frequently with PD-1 inhibitor use [4]. Clinical symptoms such as extraocular, bulbar, and limb muscle weakness, as well as respiratory difficulty, may present as an exacerbation of pre-existing MG or de novo in patients without prior MG diagnosis [4]. MG is also frequently associated with “Triple M” syndrome, which includes concurrent myositis and myocarditis, further complicating prognosis and management [4,12].

ICI-related encephalitis represents approximately 13% of nirAEs and is the most frequent CNS disorder induced by ICIs [10]. Meningoencephalitis accounts for about 50% of these cases, with focal encephalitis (including limbic, cerebellar, and brainstem forms) comprising the remaining 50% [10]. Encephalitis appears to be more frequent in lung cancer patients receiving anti-PD-L1 therapy [10]. The usual interval between initiation of ICI therapy and onset of encephalitis is approximately 12 weeks [3]. Myelitis is rare, accounting for only 2% of cases, and may occur in isolation or in association with multiple sclerosis or neuromyelitis optica spectrum disorder (NMOSD) [10]. Although uncommon, transverse myelitis has been reported following treatment with anti-PD-1 agents alone or in combination with anti-CTLA-4, often with high relapse or refractory rates [13]. Symptoms typically include paraparesis, sphincter dysfunction, tactile or thermal sensory disturbances, and proprioceptive ataxia [14]. The median onset of myelitis is around 4 months, though delayed presentations have occurred after up to 51 cycles of ICI therapy administered over approximately 45-50 months (assuming 4 weeks per cycle) [10]. Similar to encephalitis, myelitis appears to be more frequently associated with PD-L1 administration [10]. Lastly, aseptic meningitis accounts for approximately 3% of nirAEs and is more frequently associated with anti-CTLA-4 or combination (anti-PD-L1 and anti-CTLA-4) therapies [10]. The median reported time to onset of meningitis is between 2 and 3 months [10].

Unexpectedly, one study found an association between the development of irAEs and nirAEs and longer overall survival [15], whereas another reported that more than half of patients with nirAEs who survived the acute phase went on to develop chronic conditions, increasing their risk of death due to cancer progression [16]. In patients with chronic inactive nirAEs, sequelae such as cerebellar ataxia, neuromuscular weakness, vision loss, sensory disturbances, and cognitive impairment were commonly observed [16].

In this case series, we present four cases demonstrating central and/or peripheral neurotoxicity in the setting of ICI use for the treatment of thoracic and non-thoracic malignancies.

## Case presentation

Case 1

A 47-year-old woman with a history of squamous cell carcinoma of the cervix with metastases to the lungs and mediastinum, and longitudinally extensive transverse myelitis of the cervical/thoracic spine (diagnosed approximately five months before this presentation), presented to the emergency department by EMS with a two-week history of confusion progressing to altered mental status (AMS). The patient's husband had noted a sudden onset of involuntary facial movements with gaze deviation, concerning for seizure activity, and an irregular breathing pattern the night before, prompting him to call EMS. To summarize her oncologic history, she was diagnosed with squamous cell carcinoma of the cervix approximately five years earlier. She was initially treated with cisplatin infusion and radiation therapy for two months, which included nine days of brachytherapy. Around two years later, she was restarted on palliative radiation therapy with weekly paclitaxel and carboplatin but subsequently developed blue toe syndrome. She was then transitioned to maintenance pembrolizumab therapy (400 mg every six weeks) until presentation to the hospital.

**Figure 1:**
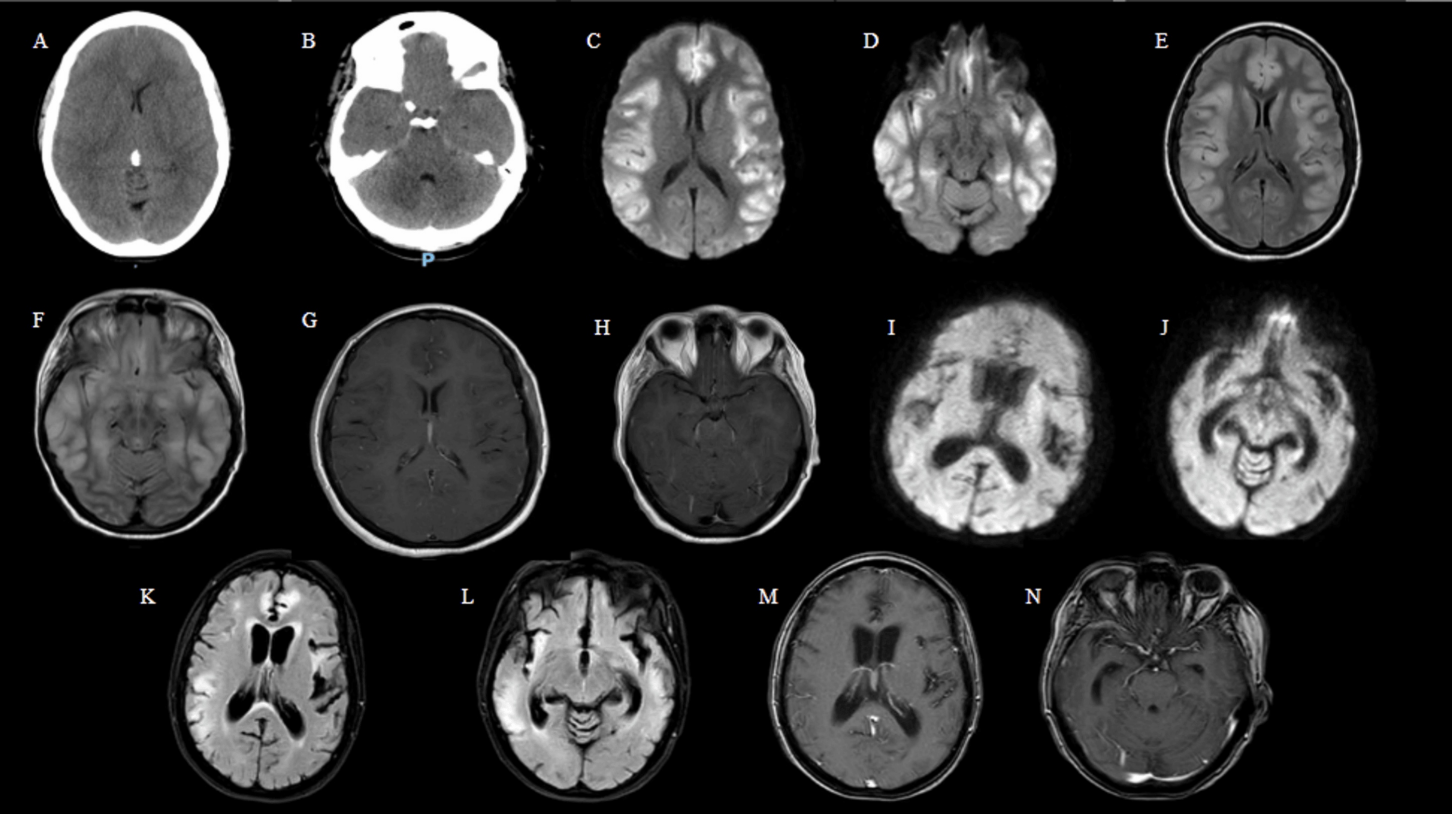
Neuroimaging findings of Case 1. (A, B) CT head scan obtained on hospital day 1, showing global cerebral edema and brainstem compression at the midbrain level; (C-H) MRI brain scan obtained on hospital day 12: (C, D) diffusion-weighted imaging (DWI) showing extensive hyperintensities in the cerebral cortex; (E, F) T2 Fluid-Attenuated Inversion Recovery (T2-FLAIR) sequence showing hyperintensity changes in the corresponding areas of DWI abnormalities; and (G, H) post-contrast axial imaging showing leptomeningeal enhancement consistent with encephalitis. (I-N) Follow-up MRI brain scan obtained on day 75 after admission: (I, J) DWI showing improvement in cortical hyperintensity changes; (K, L) T2-FLAIR sequences of corresponding sections demonstrating predominant limbic region hyperintensities with concurrent brain atrophy and ex vacuo dilatation of the lateral and third ventricles; and (M, N) post-contrast axial imaging showing improving sequelae of limbic encephalitis.

She had presented to a different hospital for AMS ten days prior and had an inconclusive workup, including an unremarkable CT scan of the head, and was discharged home. During the current hospital admission, a CT scan of the head revealed global cerebral edema with hypoattenuation of the basal ganglia, ventricular effacement, and brainstem compression (Figure 1A-1B). Due to extensive cerebral edema and increased intracranial pressure (ICP), MRI of the brain was deferred. Long-term electroencephalogram (LTM-EEG) monitoring showed diffuse slowing with epileptiform transients, and the patient was loaded with levetiracetam and maintained on a 1 g twice-daily dose. Differential diagnoses considered included infectious and autoimmune encephalitis, as well as ICI-induced neurotoxicity with encephalitis. A lumbar puncture (LP) revealed an elevated opening pressure of 30 cm of water, lymphocytic pleocytosis (corrected total nucleated cell count of 20-30 cells/mm³), and an elevated protein level (53 mg/dL). The meningitis panel, autoimmune encephalitis panel, and Herpes simplex virus PCR were negative. However, the cytokine panel showed normal but borderline interleukin-2 (IL-2) receptor levels (26.7 pg/mL; reference interval <26.8 pg/mL) and IL-6 levels (5.6 pg/mL; reference interval <7.5 pg/mL). A right frontal ICP bolt was placed for ICP monitoring, and she was treated with mannitol, 23.4% sodium chloride, high-dose propofol, and midazolam infusions to manage the ICP crisis. She was additionally started on high-dose corticosteroids for presumed ICI-related neurotoxicity syndrome and/or autoimmune encephalitis.

A repeat CT head on hospital day 5 showed worsening cerebral edema. The patient was subsequently given a standard 0.4 g/kg/day dose of intravenous immunoglobulin (IVIg) for five days for refractory irAEs, based on a literature review, and was later transitioned to a five-session PLEX therapy on hospital day 6. Given the borderline levels of interleukins, a dose of tocilizumab was added to intensify treatment. Once cerebral edema had improved, an MRI of the brain was obtained on hospital day 12, showing multifocal cortical signal abnormalities most compatible with nonspecific encephalitis (Figure 1C-1H). MRI of the spine was unremarkable for any acute changes. The hospital course was further complicated by MSSA pneumonia, which was appropriately treated with antibiotics. Given continued dependence on mechanical ventilation, a tracheostomy and a percutaneous endoscopic gastrostomy (PEG) tube were performed. She later developed paroxysmal sympathetic hyperactivity (PSH), characterized by episodes of excessive sympathetic nervous system activity, which was managed with a combination of propranolol, amlodipine, and clonazepam. Neurological examination improved slightly over the hospital course, with the patient able to follow simple central commands with intermittent delayed responses; however, tone, strength, and purposeful movement in the bilateral upper and lower extremities remained significantly diminished. She also developed marked aphasia and moderate cognitive impairment. The patient was eventually transferred to a long-term acute care (LTAC) facility on hospital day 26. Two months later, she continued to show neurological improvement, having regained antigravity strength in all extremities, was able to speak some words though aphasia persisted, and was tolerating a minced and moist diet. A repeat MRI brain on day 75 at the LTAC showed improving sequelae of limbic encephalitis with hydrocephalus ex vacuo (Figure 1I-1N). From an oncologic standpoint, no further cancer-directed treatments were available due to her current functional status and progression of disease while on pembrolizumab therapy.

Case 2

A 79-year-old man with a history of stage IVb non-small cell lung adenocarcinoma with osseous metastases presented with severe bilateral ptosis and complete bilateral ophthalmoparesis with non-reactivity to light. To summarize his cancer therapy regimen, he was started on pembrolizumab with adjuvant carboplatin and pemetrexed therapy every three weeks for the first four cycles, with plans to later transition to pembrolizumab and pemetrexed every three weeks for the next eleven cycles.

During this admission, a CT scan of the head revealed no acute intracranial abnormalities or extraocular muscle pathology, except for chronic bilateral frontal lobe infarcts. The patient had an MRI-incompatible pacemaker. He was started on IVIg therapy at a total dose of 2 g/kg over three days, along with a trial of pyridostigmine 30 mg for suspected neuromuscular junction disorder such as MG. Shortly after admission, the patient developed dysarthria and increasing respiratory difficulty. Due to worsening bilateral ptosis and decreased facial strength, treatment was escalated to five sessions of plasma exchange (PLEX) therapy in addition to high-dose corticosteroids.

A LP revealed albuminocytologic dissociation, with a protein level of 122 mg/dL (reference interval: 15-60 mg/dL) and a white blood cell count of 5 cells/mm³ (reference interval: 0-5 cells/mm³). The autoimmune antibody panel and MG antibody panel were negative. Electromyography (EMG) and nerve conduction study (NCS) revealed an autoimmune sensorimotor polyneuropathy meeting electrodiagnostic criteria for chronic inflammatory demyelinating polyneuropathy (CIDP). Pembrolizumab was held due to concern for ICI-mediated CIDP. With progression of PLEX therapy, the patient’s dysarthria improved; however, he developed new-onset hyperreflexia in the bilateral lower extremities, inconsistent with CIDP and potentially indicative of new central nervous system involvement. The hospital course was further complicated by acute ischemic necrosis of the small bowel, requiring subtotal colectomy and end ileostomy. There was minimal improvement in the neurological examination by the end of the five PLEX sessions, so corticosteroids were tapered by 10 mg per week. Subsequently, he developed worsening respiratory depression with Cheyne-Stokes breathing and dysphagia. After a family discussion, he was transitioned to comfort care. The patient died on hospital day 21.

Case 3 

An 84-year-old man with a history of stage III non-small cell lung adenocarcinoma with potential osseous metastases presented to the hospital with generalized weakness, fatigue, visual hallucinations described as “spiral-looking shadows,” and a remote history of diplopia. His cancer therapy regimen initially included radiation therapy along with six cycles of weekly carboplatin and paclitaxel. He was then transitioned to durvalumab therapy for one year.

Shortly after admission, he experienced a rapid decline in mentation, with intermittent myoclonic activity of the upper and lower extremities lasting approximately ten seconds. He was found to be obtunded the following morning. A CT scan of the head and MRI of the brain were unremarkable. LTM-EEG monitoring revealed no epileptiform activity. On neurological examination a few days later, he was noted to have limitations in bilateral adduction of the eyes with downward gaze, mild ptosis, and mildly reduced facial strength and slow speech. These symptoms followed a waxing and waning pattern, worsening as the day progressed. A trial of pyridostigmine 60 mg was initiated, which improved his speech. A five-session PLEX therapy was planned. EMG and NCS showed a non-length-dependent axonal sensorimotor polyneuropathy, which did not meet criteria for an autoimmune neuropathy. The repetitive nerve stimulation study was normal but likely confounded by concurrent pyridostigmine use. Aldolase, MG antibody panel, myomarker panel, and paraneoplastic panel were all negative. Following the PLEX sessions, he was started on a standard course of IVIg therapy for five days, followed by maintenance therapy of 1 g/kg every three weeks. He was then transferred to a LTAC facility, where his regimen was optimized with continued pyridostigmine and transition of IVIg dosing to every two weeks. At the LTAC facility, he developed respiratory failure and laryngeal edema due to suspected posterior glottic stenosis, requiring tracheostomy and gastrostomy. The family elected to pursue comfort measures. The patient passed away three months after his initial admission.

Case 4 

A 74-year-old woman with a history of thyroid cancer and malignant pleural mesothelioma, status post video-assisted thoracoscopic surgery (VATS), presented to the hospital with generalized weakness and worsening shortness of breath, suspected to be a MG crisis in the setting of a UTI.

Previously, around the time of her intrathoracic biopsy, she had developed symptoms suggestive of a neuromuscular junction disorder. Shortly thereafter, she was started on a palliative therapy regimen that included a combination of ipilimumab every six weeks with adjuvant nivolumab on days 1 and 22. Six months into therapy, her regimen was discontinued due to concerns of a seronegative MG crisis in the setting of dual ICI therapy, which is usually contraindicated in MG. She was then started on maintenance therapy for MG, including pyridostigmine 60 mg three times daily and prednisone 10 mg twice daily.

Upon admission to the critical care unit for her MG crisis, her pyridostigmine dose was increased to 90 mg every eight hours with the addition of high-dose corticosteroids. IVIg therapy at 0.4 g/kg/day was initiated for a total of five days, resulting in improvement of respiratory distress, dysphagia, and diplopia. She was discharged home with a caregiver and a recommendation for home-based physical therapy, along with IVIg maintenance therapy of 1 g/kg every four weeks for five months and prednisone 15 mg daily.

However, she continued to deteriorate, experiencing increasing fatigue and dyspnea. As her bulbar symptoms remained stable, the fatigue and dyspnea were presumed to be primarily related to her malignancy. The family opted for home hospice care, and the patient passed away one month later, approximately two months after her initial admission.

A summary of patient characteristics and their disease courses is presented in Table 1.

**Table 1:** Characteristics and disease courses of each case. AMS: Altered mental status; ICI: Immune checkpoint inhibitor; IVIg: Intravenous immunoglobulin; NM PET/CT: Nuclear medicine positron emission tomography/computed tomography; PEG: Percutaneous endoscopic gastrostomy; PLEX: Plasma exchange; SOB: Shortness of breath.

Parameter	Case 1	Case 2	Case 3	Case 4
Age/Sex	47/F	79/M	84/M	74/F
Associated Cancer	Cervical cancer with lung and mediastinal metastases	Non-small cell lung adenocarcinoma (stage IVb) with osseous metastases	Non-small cell lung adenocarcinoma (stage III) with possible osseous metastases	Thyroid cancer and malignant pleural mesothelioma
ICI Therapy	Pembrolizumab	Pembrolizumab + carboplatin + pemetrexed → pembrolizumab + pemetrexed	Durvalumab	Ipilimumab + nivolumab
Duration/Cycles of ICI Therapy	Approximately 11 cycles total over 5 months	15 cycles over 10 months	2 cycles over 2 months	6 cycles over 6 months
Time from First ICI Dose to Presentation	Approximately 17 months	Approximately 9 months	Approximately 2 months	Approximately 8 months
Presenting Symptoms	Altered mental status (AMS), involuntary movements, irregular breathing	Bilateral ptosis and ophthalmoparesis	Weakness, fatigue, visual hallucinations, diplopia	Weakness, worsening shortness of breath, myasthenia gravis exacerbation
Imaging Findings	CT Head: Global cerebral edema with hypoattenuation of the basal ganglia, ventricular effacement, and brainstem compression. MRI Brain: Multifocal cortical signal abnormalities. MRI Spine: Longitudinally extensive transverse myelitis of the cervical/thoracic spine.	CT Head: Chronic bilateral frontal lobe infarcts. MRI Brain: Not obtained (MRI-incompatible cardiac pacemaker).	CT Head: Unremarkable. MRI Brain: Unremarkable.	NM PET/CT (skull to mid-thigh): Marked interval progression of hypermetabolic pleural disease within the right hemithorax, and new metastatic upper abdominal lymph nodes.
Hospital Course / Treatment	IVIg, PLEX, high-dose corticosteroids, mechanical ventilation, tracheostomy, PEG tube	IVIg, PLEX, high-dose corticosteroids	Mechanical ventilation, IVIg, PLEX, tracheostomy, PEG tube	IVIg and corticosteroids
Final Outcome	Alive and recovering	Deceased	Deceased	Deceased

## Discussion

These four cases highlight the development of central and peripheral neurotoxicity in the setting of ICI use in cancer patients. Case 1 developed transverse myelitis, global cerebral edema, and seizures, while Cases 2, 3, and 4 developed neurological symptoms consistent with sensorimotor polyneuropathy, concerning for new-onset or acute exacerbation of pre-existing MG.

Global cerebral edema, as seen in Case 1, is often associated with the development of infectious or autoimmune encephalitis [17]. This patient’s CSF analysis was significant for lymphocytic pleocytosis with an elevated protein level. ICI-related encephalitis often manifests as pleocytosis in CSF analysis, typically combined with high protein levels in 77-98% of patients [10]. EEG findings may demonstrate seizures, temporal slowing, nonspecific patterns, or may be normal [1]. Case 1’s EEG was significant for diffuse slowing with epileptiform transients. The median time of onset of encephalitis from initiation of ICI therapy is approximately four months, with delayed onset reported after up to 51 cycles spanning 45-50 months, assuming four weeks between each cycle. Case 1 had a longer latency period of 17 months compared to the other three cases and received approximately 11 cycles of therapy. Although the likelihood of developing both low-grade and high-grade central and peripheral nirAEs decreases significantly by the second month after ICI initiation [18], it is important to recognize that the development of nirAEs may occur at any time following ICI use. Previous studies have reported complete recovery from immune-related encephalitis over several weeks, with variable timelines following ICI discontinuation and initiation of corticosteroid therapy [19]. Patients with longer recovery times often had more severe autoimmune encephalitis characterized by seizures and cognitive impairment; some experienced residual cognitive impairment, recurrent seizures, or clinical relapse [19]. Case 1 demonstrated characteristics of severe autoimmune encephalitis with seizures, significant aphasia, and moderate cognitive impairment requiring placement in a facility and life-sustaining measures such as tracheostomy and gastrostomy. A prolonged recovery period is anticipated in such a case, though long-term outcomes remain difficult to predict.

Global cerebral edema from encephalitis can also mimic cytotoxic edema observed in anoxic brain injury, seizure-related damage, or vascular injury, and is often misdiagnosed in the early stages, leading to severe complications [20]. Because treatment may differ between cytotoxic and vasogenic edema, it is important to distinguish the underlying etiology early. Given that all our patients had underlying malignancies, it is also crucial to differentiate paraneoplastic syndromes from ICI-induced central neurotoxicity as potential causes of these neurological symptoms. High-risk phenotypes such as limbic encephalitis, rapidly progressive cerebellar ataxia, and subacute sensory neuropathy are particularly suggestive of paraneoplastic neurological syndromes [21]. Paraneoplastic neurological syndromes are rare autoimmune disorders resulting from immune responses against neural antigens ectopically expressed by cancer cells [21]. Syndromes associated with antibodies against intracellular antigens (e.g., anti-Hu, anti-Ma2, or anti-Yo) usually have a less favorable prognosis compared to those involving antibodies against cell-surface antigens (e.g., anti-NMDA receptor) [21]. Molecular mimicry and cross-reactivity between tumor antigens and similar epitopes on healthy cells have been proposed as underlying mechanisms behind nirAEs [9]. Another suggested mechanism for nirAE development is epitope spreading, the release of tumor and non-tumor antigens after tissue damage, which can facilitate new immune responses and trigger autoimmunity against normal self-tissues [9]. Epitope spreading in the setting of immunosuppressive therapy may further manifest as severe neurotoxicity. Each neural antibody is closely associated with specific cancer types and clinical presentations.

Compared to central neurotoxicity, the development of MG following the first ICI use has been reported to occur at a median of approximately four weeks [22]. Cases 2 and 4 in our series had longer latency periods ranging from 2-9 months, again signifying that peripheral nirAEs can occur at any time following ICI administration. Approximately 96% of patients developing ICI-related MG require hospitalization, with 19% requiring invasive ventilation and 22% requiring non-invasive positive-pressure ventilation [22]. The prognosis of MG varies, with outcomes ranging from complete resolution (19%), partial improvement (55%), to respiratory failure (26%) without improvement despite IVIg or PLEX therapy [22].

Therapeutic recommendations for neurotoxicity related to ICIs include discontinuation of immunotherapy, administration of high-dose steroids, and consideration of intravenous immunoglobulin, plasma exchange therapy, or other immunosuppressive agents in refractory cases [9]. Discontinuation of ICI has been found to be a favorable prognostic factor for overall survival in patients receiving either ICI monotherapy or combination therapy with chemotherapy [23]. However, other studies have shown that while the majority of patients experience a durable response lasting more than six months, some develop oligoprogression that can be managed with local radiotherapy, and a smaller subset experience cancer progression within six months [24].

The safety of rechallenging patients with ICIs after a prior nirAE warrants careful risk-benefit assessment [25]. In some studies, inclusion criteria for ICI rechallenge included whether the initial regimen was a single-agent or combination ICI, whether the initial severe toxicity was graded as 3 or 4 per the National Cancer Institute Common Terminology Criteria for Adverse Events, and whether the irAE or nirAE had achieved near or complete resolution before retreatment [26,27]. Another case report describing successful ICI reinitiation emphasized the importance of a favorable initial tumor response before restarting immunotherapy [28]. Prior to ICI re-administration, the new treatment regimen, malignancy status (progression, partial response, or stability) before rechallenge, and any concurrent therapies such as radiation or chemotherapy should be carefully considered [26].

There have been reports of patients receiving subsequent ICIs after a nirAE without recurrence of the initial toxicity [25]. However, other studies have found that approximately 13% of patients experience recurrence of the same irAE, and another 13% develop new irAEs with a latency period similar to the initial event [26]. Rechallenging with ICIs has been more frequently investigated in cases of irAE development among melanoma patients, whereas fewer studies have focused on other thoracic and non-thoracic malignancies and the associated risk of nirAEs. Further exploration of how resuming ICI therapy or transitioning to another type of ICI may affect recurrence or the development of nirAEs is of significant importance to improving both overall survival and progression-free survival in cancer patients.

## Conclusions

These four cases highlight the development of central and peripheral neurotoxicity in the setting of ICI use. An increased latency period between the initial administration of ICIs and the onset of neurological irAEs was noted in all four cases, though it was more prolonged in the development of central neurotoxicity, as seen in Case 1. This emphasizes that the occurrence of neurological irAEs should be anticipated at any time following the initiation of ICI therapy. It is also important to differentiate between the various etiologies of cerebral edema and to consider paraneoplastic syndromes in patients with pre-existing malignancies to better guide management and prognosis. Variable prognoses can be expected depending on the severity of central or peripheral neurotoxicity. Lastly, ICI discontinuation is often necessary to manage nirAEs, and rechallenging ICIs should be approached with caution, underscoring the need for future studies to further assess the associated risks and benefits.
